# Pneumatosis Cystoides Intestinalis in Muscular Dystrophy and Congenital Myopathies: A Report of Five Cases

**DOI:** 10.7759/cureus.61188

**Published:** 2024-05-27

**Authors:** Yu Aihara, Eri Takeshita, Emiko Chiba, Kaoru Yamamoto, Yuko Shimizu-Motohashi, Noriko Sato, Hajime Ariga, Hirofumi Komaki

**Affiliations:** 1 Department of Child Neurology, National Center Hospital, National Center of Neurology and Psychiatry, Tokyo, JPN; 2 Department of Radiology, National Center Hospital, National Center of Neurology and Psychiatry, Tokyo, JPN; 3 Department of General Internal Medicine, National Center Hospital, National Center of Neurology and Psychiatry, Tokyo, JPN; 4 Translational Medical Center, National Center of Neurology and Psychiatry, Tokyo, JPN

**Keywords:** myotubular myopathy, radiology, congenital myopathy, muscular dystrophy, pneumatosis cystoides intestinalis

## Abstract

Pneumatosis cystoides intestinalis (PCI) is a rare disease wherein air accumulates in the intestinal subserosa and submucosa, causing multiple gaseous cysts within the gastrointestinal wall. While PCI has various known risk factors, reports identifying muscular diseases as a factor are scarce. The aim of this study is to elucidate the clinical characteristics of PCI in muscle disease. We present a case series of five cases, including two cases of Duchenne muscular dystrophy (DMD) and three cases of rare congenital myopathies. All cases are of male patients, with poor intestinal peristalsis and constipation, who underwent tube feeding and mechanical ventilation via tracheostomy. They had no signs of severe complications, such as intestinal necrosis, and all of them improved with conservative treatment. Case 1 is a 23-year-old man with DMD who developed cardiopulmonary arrest at the age of 20 years. Pulmonary hemorrhage occurred three months before the incidental detection of PCI in the ascending colon, which resolved with conservative oxygen treatment. Case 2 is a 25-year-old man with DMD who progressed to immobility necessitating tracheostomy at the age of 20 years. He experienced persistent abdominal pain and nausea, and PCI was detected in the cecum and ascending colon. He showed near-complete resolution of PCI after three months of conservative treatment. Case 3 is a six-year-old boy with reducing body myopathy. Constipation was diagnosed at four years of age. He experienced intermittent bloody stools, leading to the incidental detection of PCI at six years of age. After two months of conservative treatment, the PCI resolved with no subsequent recurrence. Case 4 is a 33-year-old man with infantile severe myotubular myopathy. He required mechanical ventilation immediately after birth and later underwent tracheostomy and tube feeding due to complications. At the age of 27 years, PCI was incidentally detected on abdominal CT. He had episodes of remission and worsening for a few years; however, PCI completely resolved after three years. Case 5 is a 27-year-old man with nemaline myopathy. At the age of 14 years, he had persistent bloody stools. After lower gastrointestinal endoscopy, he was diagnosed with PCI with numerous rectal cysts. PCI required no specific therapeutic intervention. There was spontaneous resolution of PCI and bloody stools. Given that PCI lacks specific symptoms and cases with muscular diseases often experience abdominal issues, many cases are liable to be overlooked or misdiagnosed. Cases with muscular diseases complaining of persistent abdominal symptoms should undergo radiographic imaging to rule out PCI.

## Introduction

Pneumatosis cystoides intestinalis (PCI) is characterized by multiple gaseous cysts within the gastrointestinal wall caused by air accumulation in the intestinal subserosa and submucosa [1,2]. PCI is a rare disease with an estimated incidence of approximately 0.03% [3], and its symptoms, such as abdominal pain, diarrhea, distention, nausea and vomiting, bloody stool, mucous stool, and constipation, are considered nonspecific [2]. Clinical diagnosis of PCI is sometimes difficult, and diagnosis is generally made by X-ray, computed tomography (CT), endoscopy, or surgery [2,4-6]. On both X-ray and CT scanning, PCI typically presents as a low-density linear or bubbly gas pattern within the bowel wall [1,4,6]. The morphological indicators of gas formation in the bowel wall can be categorized as follows: (i) cystic or bubbly, characterized by isolated air bubbles, or (ii) curvilinear or linear, characterized by continuous air bands within the bowel wall [1].

Although PCI has many reported risk factors [1,4,7,8], no reports on muscular diseases as a risk factor are available. PCI has been reported in dermatomyositis [9], polymyositis [10], and myotonic dystrophy [11], but not in other muscular diseases. Here, we report two cases of Duchenne muscular dystrophy (DMD) and three cases of rare congenital myopathies, all exhibiting PCI.

## Case presentation

**Figure 1:**
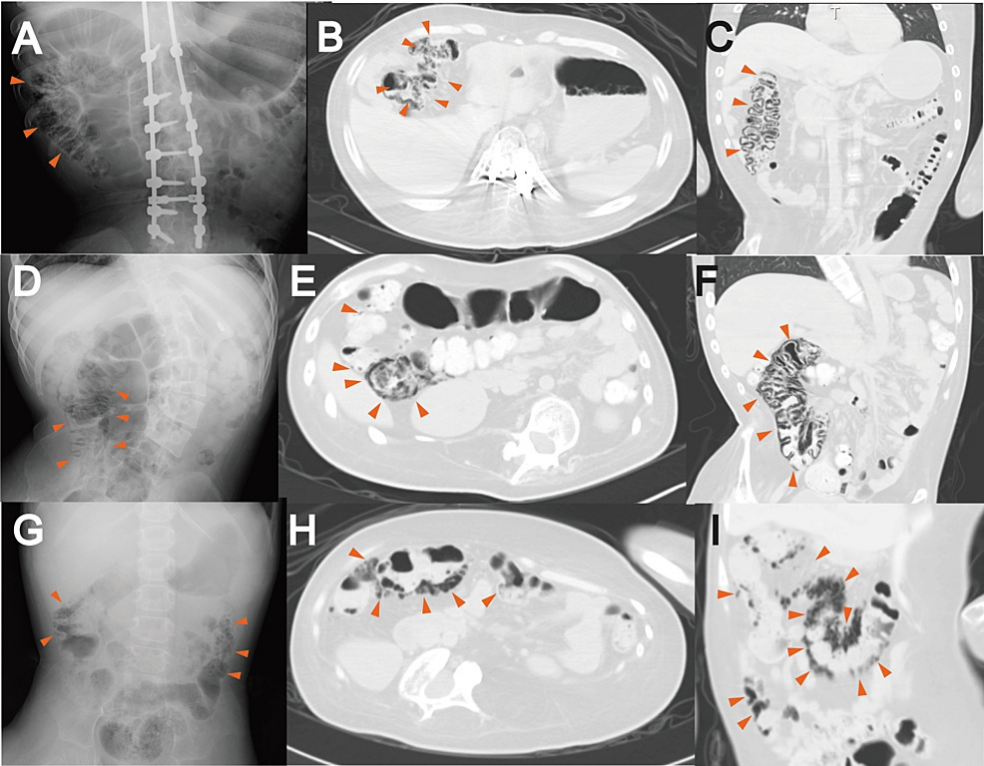
Abdominal radiographs and computed tomography (CT) images of the cases (A–C) Case 1, (D–F) case 2, (G) case 3, and (H–I) case 4. (A), (D), and (G) Abdominal X-ray. (B), (E), and (H) Axial CT scan images of lung window. (C), (F), and (I) Coronal CT scan images of lung window. Linear gas shadows were observed (red arrowheads).

We present a case series of five cases with PCI of which two cases had DMD and one case each had reducing body myopathy, infantile severe myotubular myopathy, and nemaline myopathy, respectively (Table 1). Abdominal radiographs and computed tomography images of cases 1-4 are presented in Figure 1.

**Table 1:** Clinical information of cases Xp, pain radiograph; CT, computed tomography; PCI, pneumatosis cystoides intestinalis; DMD, Duchenne muscular dystrophy; PIP, peak inspiratory pressure; PEEP, positive end expiratory pressure; PS, pressure support; FiO_2_, fraction of inspired oxygen; IPAP, inspiratory positive airway pressure; EPAP, expiratory positive airway pressure; Ti, inspiratory time; PEG, percutaneous endoscopic gastrostomy; NA, not available

Disease Category	Muscular Dystrophy		Congenital Myopathies		
Case No.	1	2	3	4	5
Diagnosis	DMD	DMD	Reducing body myopathy	Myotubular myopathy	Nemaline myopathy
Molecular diagnosis	DMD, exon49-50 deletion	DMD, exon3-13 deletion	FHL1, c.386G>A, p.C129Y	Not tested	Not tested
Sex (Male =M/Female=F)	M	M	M	M	M
Age at onset	23	20	6	27	14
Age at the last evaluation	23	25	6	33	27
Complaint at onset	Abdominal distension, tenesmus	Abdominal pain (right lateral to right lower), nausea	Abdominal pain	No	Bloody stool
Xp	Yes	Yes	Yes	Yes	No
CT	Yes (ascending colon)	Yes (cecum and ascending colon)	No	Yes (transverse colon)	No
Portal vein gas	No	No	NA	No	NA
Abdominal free air	No	No	NA	No	NA
Endoscopy	No	No	No	No	Yes (rectum)
Treatment	Oxygen supplementation	Oxygen supplementation	No	No	No
Prognosis	Improved	Improved	Improved	Improved	Improved
Tube feeding	Yes (gastrostomy)	Yes (gastrostomy)	Yes (gastrostomy)	Yes (nasogastric tube)	Yes (nasogastric tube)
Mechanical ventilation	Yes (tracheostomy)	Yes (tracheostomy)	Yes (Tracheostomy)	Yes (Tracheostomy)	Yes (Tracheostomy)
Mode and setting	PIP (above PEEP) 13, PEEP 5, PS 8, f 12, FiO2 0.21	S/T, IPAP 12, EPAP 4, f 12, Ti 1.2, FiO2 0.21	S/T, IPAP 24, EPAP 5, f 20, Ti 1.0, FiO2 0.21	T, IPAP 22, EPAP 4, f 16, Ti 1.0	T, IPAP 22, EPAP 4, f 14, FiO2 0.21
Glucocorticoid	Yes (5 mg/day)	Yes (5 mg/2days)	No	No	No
Other medications at risk for PCI	No	No	No	No	No
Pulmonary diseases	Yes (pulmonary hemorrhage)	No	No	No	No
Gastrointestinal diseases	Yes (constipation, poor intestinal peristalsis, suspect of inflammatory bowel disease)	Yes (constipation, poor intestinal peristalsis, Ulcerative colitis)	Yes (constipation, poor intestinal peristalsis)	Yes (constipation, poor intestinal peristalsis)	Yes (poor intestinal peristalsis)
Diabetes mellitus	No	No	No	No	No
Autoimmune diseases	No	No	No	No	No
Infectious diseases	No	No	No	No	No
Cancer	No	No	No	No	No
Organ transplantation	No	No	No	No	No
Surgery before onset	Yes (gastrostomy by PEG, tracheostomy)	Yes (gastrostomy by PEG, tracheostomy)	Yes (gastrostomy by PEG, tracheostomy)	Yes (tracheostomy)	Yes (tracheostomy)
Other complications	Yes (anxiety disorders)	No	No	No	No

Case 1

Case 1 is a 23-year-old man with DMD. The case has a family history of DMD and was diagnosed genetically with DMD exon 49-50 deletion at 1 year of age. He has been taking prednisolone at 5 mg/day since the age of six years. At 20 years, he choked on phlegm and experienced cardiopulmonary arrest but was eventually resuscitated. Although his intellectual level was retained, he could not move, except his facial muscles; he was managed with gastrostomy and ventilation through a tracheotomy. He had been hospitalized since the age of 21 years. At 23 years of age, he had frequent abdominal distension, tenesmus, and sometimes, nausea and vomiting. He was then diagnosed with decreased bowel sounds and constipation. Pulmonary hemorrhage also occurred three months before the detection of PCI and thrice thereafter. His abdominal X-ray and CT showed incidental PCI in the ascending colon, with no evidence of intestinal ischemia or portal gas (Figures 1A-1C). He was treated conservatively with oxygen at a FiO_2_ of 0.3, and two months later, the PCI resolved.

Case 2

Case 2 is a 25-year-old man with DMD. He had no family history of DMD and no abnormalities three years of age, when he had difficulty climbing stairs, and at six years of age, elevated serum CPK (19,951 IU/ml) was noted. The case was referred to our hospital, where genetic testing via multiplex ligation-dependent probe amplification was done and a deletion in DMD exon 3-13 was identified. Thus, he was diagnosed with DMD. He has been taking prednisolone at 15 mg/day since the diagnosis. He was diagnosed with constipation at the age of 18 years. Later, he could not walk, and owing to repeated aspiration pneumonia and difficulty swallowing, he underwent tracheostomy and had been on mechanical ventilation and tube feeding since the age of 20 years. He had been hospitalized since then. Shortly after the admission, he had persistent right lateral to right lower abdominal pain and nausea. The symptoms did not improve for more than a week, and abdominal X-ray and CT revealed PCI in the cecum and ascending colon (Figures 1D-1F). A small amount of free air was also detected around the ascending colon, but there was no evidence of intestinal ischemia or portal gas. The case was treated conservatively, and three months later, the PCI almost disappeared. However, he had bloody stools at the age of 22; through endoscopy, he was diagnosed with ulcerative colitis. The case has been treated with sulfasalazine and elemental nutrition. Subsequently, PCI has not recurred.

Case 3

Case 3 is a six-year-old boy with reducing body myopathy. He had no family history and no abnormalities until one year of age, when his head control became unstable and showed Gowers’ sign while standing up. At the age of 18 months, his muscle biopsy, which was conducted at our hospital, revealed numerous reduced bodies and identified a hemizygous variant c.386G > A:p.(C129Y) in FHL1. At the age of two years, cardiopulmonary arrest occurred because of mechanical ventilation abnormality. He was resuscitated and required tube feeding and mechanical ventilation through tracheostomy. He had been admitted to our hospital at the age of four years. He was diagnosed with constipation at four years of age. He presented with bloody stools every few months and underwent routine abdominal X-rays. At the age of six years, abdominal X-ray showed incidental PCI in the ascending colon, but with no evidence of free gas or intestinal ischemia (Figure 1G). He received no specific therapeutic intervention, only follow-up imaging, and improved. After two months, the PCI resolved with no subsequent recurrence.

Case 4

Case 4 is a 33-year-old man with infantile severe myotubular myopathy. He had no definite history, although his elder brother died of an unknown cause shortly after birth. This case did not establish spontaneous respiration immediately after birth, requiring mechanical ventilation via endotracheal intubation in the neonatal intensive care unit. At the age of three months, he was diagnosed with myotubular myopathy through muscle biopsy. At the age of one year, owing to endotracheal tube abnormality, he had hypoxic encephalopathy and underwent tracheostomy and tube feeding. He was diagnosed with constipation at 22 years of age. At the age of 27 years, his abdominal CT scan showed incidental PCI in the ascending colon, but with no evidence of free gas, intestinal ischemia, and portal vein gas; thus, he received no specific therapeutic intervention, only follow-up imaging and improved (Figures 1H, 1I). Remission and worsening occurred for a few years, but after three years, PCI almost completely resolved.

Case 5

Case 5 is a 27-year-old man with nemaline myopathy. He had poor feeding since birth, thereby introduced to tube feeding. His respiratory condition was also unstable; hence, he underwent oxygen and airway management. However, he was placed on artificial ventilation and underwent tracheotomy at the age of three and nine months, respectively. He had been hospitalized since three years of age. At the age of five years, he had hypoxic encephalopathy resulting from ventilator problems. He was diagnosed with constipation at the age of 13 years. At the age of 14 years, he had persistent bloody stools; lower endoscopic examination revealed scattered cystoid nodules in the rectus walls, and he was diagnosed with PCI with numerous rectal cysts. Biopsy of the colon and rectal mucosa showed torsion and bifurcation of some glandular ducts and a chronic inflammatory cell infiltrate containing eosinophils. The PCI required no specific therapeutic intervention, and it was resolved spontaneously without bloody stools.

## Discussion

We present five cases of PCI in muscular diseases in this case series. To our knowledge, this report is the first to present PCI in various muscular disorders. All cases were male and had decreased bowel sounds or constipation, and they received mechanical ventilatory management with tracheostomy and tube feeding such as gastrostomy. Nevertheless, the gaseous cysts were resolved through conservative treatment, such as oxygen administration, without requiring surgery. Cases with a muscular disease often have multiple risk factors for PCI, and the actual number of PCI cases may be considerably higher.

Muscular dystrophy and myopathy are risk factors for PCI. The hypotheses for PCI etiology include gastrointestinal dysmotility, inflammation, intestinal mucosa damage, pulmonary damage, and dysbacteriosis [4,5,12]. According to the five cases presented in this study, gastrointestinal dysmotility is the most possible cause of PCI. All cases had poor intestinal peristalsis and constipation. Several risk factors for PCI have been reported, and many of them apply to the present cases [13]. All cases were on ventilatory management with positive end-expiratory pressure and experienced decreased bowel sounds and constipation [13]. Cases 1 and 2 were on corticosteroids [14]; case 1 had recurrent pulmonary hemorrhages of unknown causes and was suspected of having irritable bowel syndrome, and case 2 was endoscopically diagnosed with ulcerative colitis [15]. In a systematic analysis of 239 cases with PCI, the male-to-female ratio was 2.4:1, and the mean age at onset was 45.3 ± 15.6 years [2]. Of the cases we identified, DMD and myotubular myopathy are X-linked and predominantly male, resulting in a male predominance in this case series. Their PCI onset was before the age of 30, which indicates that PCI can develop early in muscular disease and that the prognosis is favorable because of the onset at a younger age [11]. PCI has been reported in dermatomyositis [9], polymyositis [10], and myotonic dystrophy [11], but these muscular diseases were not identified in our hospital.

Two of the five cases had DMD, which is known for its high incidence of intestinal complications [16-18]. The most common intestinal complication is constipation, which occurs in 31.7%-46.7%, followed by obesity, gastroesophageal reflux disease, inflammatory bowel disease [16,17,19], and other conditions that are considered risk factors for PCI. In a mouse model of DMD, the lack of dystrophin caused a decrease in colonic smooth muscle contractility, peristalsis, and gastrointestinal transit [20]. These data predict a higher risk of developing PCI in cases with DMD. However, PCI in DMD has not yet been reported.

Many cases may have been overlooked because of the rare incidence of PCI and its nonspecific symptoms. In previous reports, 90.8% of cases with PCI had symptoms such as abdominal pain (53.9%), diarrhea (53.0%), distention (42.4%), nausea and vomiting (14.3%), bloody stool (12.9%), mucous stool (12.0%), and constipation (7.8%) [2]. Cases with muscular diseases often have other abdominal symptoms, such as constipation, and many cases can be missed or misdiagnosed [16,17,19].

This report is a single-center, retrospective study, and the timing of testing, methods of evaluation, and treatment were not standardized. The incidence of PCI may be higher in our hospital than in other facilities because the rates of mechanical ventilation with tracheostomy or tube feedings vary by country and facility. However, as mentioned above, cases with muscular diseases have many risk factors for PCI, and many cases are liable to be missed or misdiagnosed. PCI diagnosis is generally made by X-ray, CT, colonoscopy, and surgery [2,13], but in cases with muscular diseases, the latter two are often difficult to perform immediately because of respiratory and cardiac complications. Therefore, radiographic imaging, including CT scanning, is important for diagnosing PCI in muscular diseases [13]. Aggressive and routine radiological examinations are recommended to detect and accumulate cases of PCI in muscular diseases.

## Conclusions

We presented five cases of PCI in muscle disease. The occurrence of PCI in muscle disease may be more common than expected. Owing to the nonspecific symptoms of PCI, its diagnosis is liable to be overlooked. Cases with muscular diseases complaining of persistent abdominal symptoms should undergo radiographic imaging or endoscopic studies to rule out PCI.
